# The association between plasma and MRI biomarkers in dementia with lewy bodies

**DOI:** 10.1186/s13195-025-01848-x

**Published:** 2025-08-22

**Authors:** Carmen Peña-Bautista, Katharina Bolsewig, Maria C Gonzalez, Nicholas J Ashton, Dag Aarsland, Henrik Zetterberg, Eric Westman, Olivier Bousiges, Frederic Blanc, Charlotte E Teunissen, Afina W Lemstra, Consuelo Cháfer-Pericás, Miguel Baquero, Daniel Ferreira

**Affiliations:** 1 Division of Clinical Geriatrics, Center for Alzheimer Research, Department of Neurobiology, Care Sciences and Society, Karolinska Institutet, NEO floor 7th, Stockholm, Huddinge, 141 83 SE Sweden; 2 Alzheimer’s Disease Research Group, Instituto de Investigación Sanitaria La Fe, Valencia, Spain; 3 Neurochemistry Laboratory, Laboratory Medicine Department, Amsterdam Neuroscience, VU University Medical Centers, Amsterdam UMC, Amsterdam, The Netherlands; 4 Department of Quality and Health Technology, University of Stavanger, Stavanger, Norway; 5 The Norwegian Centre for Movement Disorders, Stavanger University Hospital, Stavanger, Norway; 6 Centre for Age-Related Medicine, Stavanger University Hospital, Stavanger, Norway; 7 Department of Psychiatry and Neurochemistry, Sahlgrenska Academy, University of Gothenburg, Mölndal, Sweden; 8 Department of Old Age Psychiatry, King’s College London, London, UK; 9 Clinical Neurochemistry Laboratory, Sahlgrenska University Hospital, Mölndal, Sweden; 10 Department of Neurodegenerative Disease, UCL Institute of Neurology, London, UK; 11 UK Dementia Research Institute at UCL, London, UK; 12 Hong Kong Center for Neurodegenerative Diseases, Hong Kong, China; 13 Wisconsin Alzheimer’s Disease Research Center, University of Wisconsin School of Medicine and Public Health, Madison, USA; 14 GeRMINED Division, Service of Gerontology Mobile-Neuro-Psy-Research, University Hospitals of Strasbourg, CM2R (Research and Resources Memory Centre), Strasbourg, France; 15 University of Strasbourg and CNRS, ICube Laboratory UMR 7357 and FMTS (Fédération de Médecine Translationnelle de Strasbourg), IMIS Team, Strasbourg, France; 16 Alzheimer Center Amsterdam, Amsterdam UMC, Amsterdam, the Netherlands; 17 Division of Neurology, Hospital Universitari I Politècnic La Fe, Valencia, Spain; 18 Facultad de Ciencias de la Salud, Universidad Fernando Pessoa Canarias, Las Palmas, Spain

**Keywords:** DLB, MRI, Blood biomarkers

## Abstract

**Background:**

The diagnosis of Dementia with Lewy Bodies (DLB) is primarily based on clinical features. The main driver of DLB is alpha-synuclein-related pathology, but cerebrovascular disease (CVD) and Alzheimer’s Disease (AD) co-pathologies are often found in patients with DLB. Fluid biomarkers and magnetic resonance imaging (MRI) can provide mechanistic and diagnostic information beyond clinical features. Therefore, the aim of this study was to investigate the association of plasma biomarkers (GFAP, NfL, Aβ42/40, pTau231, pTau181) with MRI markers of neurodegeneration and CVD in DLB and in patients with AD as a control group. We also evaluated the ability of biomarkers and clinical features to discriminate between DLB and AD.

**Methods:**

We included 134 patients from the European DLB consortium (DLB (*n* = 92) and AD (*n* = 43)) with plasma biomarkers determined with Simoa and MRI assessed with radiological scales for medial temporal lobe atrophy (MTA), global cortical atrophy scale – frontal subscale (GCA-F), posterior atrophy (PA), and cerebrovascular disease (Fazekas scale). Associations between plasma and MRI biomarkers were assessed with the Mann-Whitney *U* test, and group differences and the discrimination between DLB and AD were assessed with ANCOVA, Random Forest, and ROC analyses.

**Results:**

In DLB, plasma concentrations of GFAP and NfL were associated with MTA, GCA-F, and Fazekas scale; and the Aβ42/40 ratio was associated with PA and Fazekas. Most of these associations were not statistically significant in AD. Individually, plasma and MRI biomarkers had a limited ability to discriminate DLB from AD. Plasma biomarkers helped increase the low specificity of core clinical features from 68% up to 79%, keeping the high sensitivity of 90%.

**Conclusions:**

Plasma biomarkers of AD co-pathology, glial processes and unspecific neurodegeneration are associated with MRI biomarkers of atrophy and cerebrovascular disease in DLB patients. Plasma biomarkers increase the ability of core clinical features to discriminate between DLB and AD.

**Supplementary Information:**

The online version contains supplementary material available at 10.1186/s13195-025-01848-x.

## Background

Dementia with Lewy Bodies (DLB) is a neurodegenerative dementia primarily caused by intracellular deposits of the misfolded protein alpha-synuclein [1]. Other common causes of dementia are Alzheimer’s disease (AD) and cerebrovascular disease (CVD) [2]. Importantly, patients with DLB may also have AD and CVD co-pathology in their brains, which complicates the diagnosis of DLB [3].

The diagnosis of DLB mostly relies on core clinical features (cognitive fluctuations, visual hallucinations, parkinsonism, and rapid eye movement sleep behaviour disorder (RBD)) and indicative biomarkers (e.g. ^123^Iodine-metaiodobenzylguanidine (MIBG) myocardial scintigraphy and Dopamine transporter Scan (DaTSCAN) [4]. However, structural imaging such as magnetic resonance imaging (MRI) and computed tomography (CT) is also commonly used in the differential diagnosis between DLB and AD [5], and is cheaper and more widely available than MIBG and DaTSCAN. A differential distribution of MRI- or CT-based atrophy patterns has been described in AD and DLB [6]. These patterns include typical AD, limbic-predominant, hippocampal-sparing, and the minimal atrophy pattern. It has been suggested that part of the clinical heterogeneity in neurodegenerative dementias may depend on these atrophy patterns and the presence of co-pathologies [7].

In biofluids, AD co-pathology can be assessed in-vivo with cerebrospinal fluid (CSF) and plasma biomarkers. Previous studies reported associations of CSF biomarkers of AD (amyloid β (Aβ) and phosphorylated tau (pTau) levels) with brain atrophy, clinical symptoms, and cognitive decline in DLB [8, 9]. Furthermore, abnormal CSF concentrations of AD biomarkers have been associated with higher brain alpha-synuclein pathology [10]. In recent years, the emergence of plasma biomarkers has revolutionized the diagnostic opportunities in AD. Several studies have assessed the association between plasma biomarkers and neurodegeneration assessed with MRI in AD [11]. Specifically, a lower plasma Aβ42/40 ratio was significantly associated with greater medial temporal atrophy (MTA) assessed with a visual rating scale on MRI in patients with AD [12]. Other studies have also demonstrated a significant association between higher levels of pTau181 and brain atrophy (greater cortical thinning and grey matter loss) in patients with AD [11, 13]. Apart from core plasma AD biomarkers, glial fibrillary acidic protein (GFAP) and neurofilament light (NfL) are proteins found in the astroglia and neuronal axons respectively and, therefore, are considered biomarkers of glial activation or axonal damage [14, 15]. Higher plasma levels of GFAP and NfL have been associated with more MTA in Aβ-positive patients with subjective cognitive impairment, mild cognitive impairment and dementia, as well as with CVD in incident dementia [16, 17]. A recent study showed that plasma concentrations of GFAP, NfL, Aβ42/40, and pTau181 predicted positivity in amyloid positron emission tomography (PET) in DLB patients [18]. In addition, higher plasma concentrations of GFAP, NfL and pTau181 and 231 have been associated with greater cognitive decline, and higher NfL with a higher frequency of parkinsonism in DLB [19, 20]. Elucidating the association of plasma biomarkers with MRI measures of neurodegeneration and CVD in DLB, and determining the ability of plasma and MRI biomarkers to discriminate DLB from AD is still needed to inform the mechanisms driving the neurodegenerative process in DLB and understand how to translate that knowledge to the clinical routine for the discrimination between DLB and AD.

The aims of this study were to investigate the association of plasma biomarkers of pathological mechanisms involved in neurodegeneration (amyloid, beta, Tau, inflammation and glia activation) with biomarkers of brain atrophy and vascular pathology in DLB. We also investigated the ability of plasma and MRI biomarkers to discriminate DLB from AD on their own and in combination with core clinical features. We chose to use visual rating scales that, although less sensitive than automated volumetric MRI approaches, are more robust to variation in image acquisition parameters and scanner characteristics in multi-center studies. Specifically, we selected a combination of validated rating scales that together offer a global assessment of neurodegeneration and cerebrovascular co-pathology: the MTA scale for neurodegeneration in medial temporal lobes [21], the global cortical atrophy scale – frontal subscale (GCA-F) for neurodegeneration in frontal lobe [22], and the posterior atrophy (PA) scale for neurodegeneration in parietal and occipital lobes [23], as well as the Fazekas scale for white matter signal abnormalities [24].

## Methods

### Participants and sample collection

We searched the E-DLB database for all DLB and AD patients with plasma biomarkers and MRI data available [20, 25]. A total of 134 patients from 2 E-DLB centres were available, of which 92 had a clinical diagnosis of probable DLB and 43 had a clinical diagnosis of probable AD [4]. Diagnoses were established by licensed neurologists based on detailed medical history and clinical examinations, including physical, neurologic, and psychiatric examinations [4]. DLB core clinical features of visual hallucinations, parkinsonism, and cognitive fluctuations were assessed following the International Consensus criteria, and the Mini-Mental State Examination (MMSE) was recorded as a measure of global cognitive impairment [4, 26]. 

### Plasma biomarkers

Plasma concentrations of GFAP, NfL, Aβ42 and Aβ40 were determined using the commercially available Neurology 4-Plex E kit on the Simoa HD-X Analyzer (Quanterix, USA). These biomarkers were analyzed centrally at the Neurochemistry Laboratory of Amsterdam University Medical Centers (Netherlands), as previously described in Bolsewig et al. [19]. Additionally, plasma concentrations of pTau231 and pTau181 were determined using a clinically validated in-house single molecule array (Simoa) centrally at the Clinical Neurochemistry Laboratory, Sahlgrenska University Hospital, Mölndal (Sweden), as previously described in Gonzalez et al. [20].

Plasma biomarkers Aβ40, Aβ42, GFAP and NfL were measured across three analytical runs in singlicate. Internal quality control samples were included in each run. Repeatability and intermediate precision were determined using three QC samples and showed coefficients of variation below 15% for all biomarkers, as previously described by Bolsewig et al. and González et al. [19, 20]. Specifically, repeatability CVs ranged from 2.7 to 8.2%, and intermediate precision CVs from 3.3 to 9.7%, depending on the analyte. For plasma biomarkers p-tau181 and p-tau231, all measurements were performed using a single batch of reagents, and analysts were blinded to clinical information. The analytical variation was evaluated by internal quality control samples across the study. These procedures reduced the risk of inter-batch variability and ensured high analytical consistency.

### MRI biomarkers

Brain MRI scans were obtained using different scanners and acquisition protocols. To accommodate variability across scanners and protocols, we performed a centralized assessment of scans at Karolinska Institutet (Sweden) using visual rating scales as described previously (Ferreira et al., 2017). Visual ratings were performed on T1-weigthed images using three well-stablished scales: MTA scale [21], GCA-F subscale [22], and PA scale [23]. Additionally, we investigated white matter signal abnormalities as a well-stablished marker of CVD, using the Fazekas scale on FLAIR images [24]. MTA scores range from 0 (no atrophy) to 4 (severe atrophy) and were dichotomized as abnormal using age-adjusted cut off values. Specifically, for age ranges 45–64, 65–74, 75–84, and 85–94 years, the corresponding cut off values were MTA scores ≥ 1.5, ≥ 1.5, ≥ 2, ≥2.5, respectively [27]. These age-specific cut off values were based on normative data from 345 cognitively healthy individuals and are widely used to establish abnormality in memory clinic populations [28, 29]. GCA-F and PA scores range from 0 (no atrophy) to 3 (severe atrophy). GCA-F and PA were dichotomized as normal (0 score) and abnormal (scores ≥ 1), based on prior evidence that age adjustments do not improve their diagnostic performance [28]. This approach, supported by previous studies using the same E-DLB dataset [6, 29], allows for the reliable classification of visual ratings into normal/abnormal without dedicating a specific control group within the study. Fazekas scores range from 0 (no changes) to 3 (highest degree of white matter changes), and there were dichotomized as normal when scores were < 2 and abnormal when ≥ 2, as in previous studies [30]. The four atrophy patterns described in previous studies (typical AD, limbic-predominant, hippocampal-sparing, and minimal-atrophy) were defined as a combination of the scores from MTA, GCA-F, and PA [6]. Typical AD was defined as abnormal MTA, abnormal PA and/or abnormal GCA-F. Limbic-predominant was defined as abnormal MTA, normal PA and GCA-F. Hippocampal-sparing was defined as abnormal PA, and/or abnormal GCA-F, and normal MTA. Minimal-atrophy was defined as normal MTA, PA, and GCA-F.

One single expert neuroradiologist rated all the images, blinded to any demographic or clinical information. The neuroradiologist has an excellent intra-rater reliability, with a weighted κ of 0.94 and 0.89 for MTA in left and right hemispheres respectively, 0.83 for GCA-F and 0.88 for PA in 120 random cases [27].

### Statistical analyses

Group differences for demographic and clinical variables were analysed with the Man-Whitney *U* test for quantitative variables and the Chi-Square test for categorical variables (sex and years of education).

The association between plasma biomarkers and MRI was assessed with the Mann-Whitney *U* test with dichotomized MRI ratings as the independent variable (normal vs. abnormal) and the continuous measures of plasma biomarkers as the dependent variables, separately for DLB and AD groups.

For the study of plasma biomarkers across atrophy subtypes, we used the Kruskal-Wallis test with the Bonferroni correction for post hoc paired-wise comparisons. Effect sizes were estimated from the non-parametric Mann-Whitney *U* value, considering 0.2 as a small effect, 0.5 as medium, and 0.8 as large [31].

Differences between DLB and AD groups for dichotomized MRI visual ratings (MTA, GCA-F, PA, Fazekas) and continuous plasma biomarkers (GFAP, NfL, Aβ42/40, pTau231, pTau181) were evaluated in two steps. First, group differences were tested with Mann-Whitney *U* (plasma biomarkers) and Chi-Square (MRI biomarkers) tests. Second, these analyses were followed by ANCOVA correcting for age and MMSE.

We also performed ROC analyses to evaluate the ability of plasma and MRI biomarkers to discriminate DLB from AD. These analyses were first performed separately for each individual plasma and MRI biomarker without any correction and next, corrected for age and MMSE with the unstandardized residuals obtained from the ANCOVAs described above. We finally analysed the ability of plasma and MRI biomarkers to discriminate DLB from AD in models combining different types of measures. Specifically, we performed Random Forest analysis including combinations of plasma biomarkers, MRI biomarkers, demographic measures (age, sex, years of education), MMSE, and clinical features (visual hallucinations, parkinsonism, and cognitive fluctuations) as predictors and diagnostic group (DLB vs. AD) as the outcome variable. In these Random Forest models, we report the proportion of importance (Prop imp) as a standard measure of the contribution of each variable to the model´s prediction. Higher prop imp values mean a greater contribution of the variable to the model’s prediction. We also report sensitivity and specificity values separately for each ROC analysis and Random Forest models.

All statistical analyses were performed with the SPSS software v.23 and R Studio 4.1.1. Results were considered significant when *p* ≤ 0.05.

## Results

### Cohort characteristics

Table 1 describes the key demographic and clinical characteristics of the study participants. There were no statistically significant differences between DLB and AD groups for sex and years of education. DLB patients were significantly younger and the MMSE was significantly higher in DLB, although effect sizes of these differences were small (< 0.2). Group differences in MMSE were no longer significant when controlling for age (*p* = 0.228).

**Table 1:** Key demographic and clinical characteristics of the study participants

Variable		DLB (*n* = 92)	AD (*n* = 43)	*p*-value
Age, years, median (IQR)		71 (63, 79)	75 (69, 81)	**0.034**
Sex, female, n (%)		32 (35%)	18 (42%)	0.428
Years of education, n (%)	< 9	18 (21%)	7 (17.5%)	0.921
	9–11	26 (31%)	14 (35%)	
	12–13	9 (11%)	5 (12.5%)	
	> 14	32 (38%)	14 (35%)	
MMSE, median (IQR)		25 (23, 28)	24 (22, 27)	**0.044**

Group differences in MMSE were no longer significant when controlling for age (*p* = 0.228). Bold denotes statistically significant results (*p* < 0.05). Abbreviations, DLB: Dementia with Lewy Bodies; AD: Alzheimer Disease; IQR: Interquartile range; MMSE: Mini-Mental State Examination

### Associations between plasma and MRI biomarkers

**Fig. 1:**
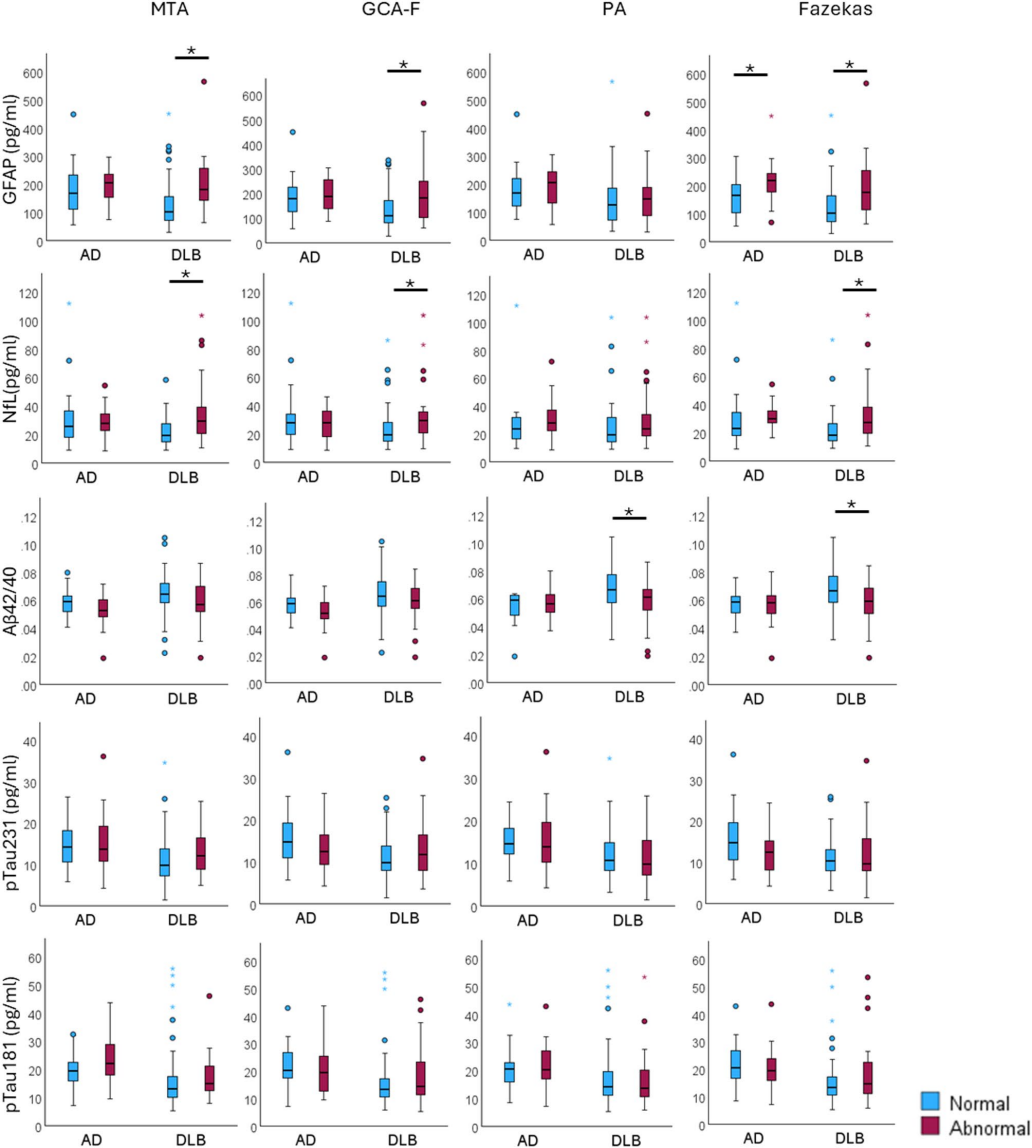
Box plots for levels of plasma biomarkers in DLB and AD patients stratified by normal vs. abnormal MRI visual ratings. *: p-value < 0.05 (Mann-Whitney test). Abbreviations, MTA: medial temporal lobe atrophy; GCA-F: global cortical atrophy scale – frontal subscale; PA: posterior atrophy; DLB: Dementia with Lewy Bodies; AD: Alzheimer Disease; GFAP: glial fibrillary acidic protein; NfL: neurofilament light; Aβ42/40: amyloid β 1–42/ amyloid β 1–40 ratio; pTau231: phosphorylated Tau 231; pTau181: phosphorylated Tau 181

Table 2 shows the associations between plasma (GFAP, NfL, Aβ42/40, pTau231, pTau181) and MRI biomarkers (MTA, GCA-F, PA, Fazekas) in DLB and AD, separately. Boxplots for all comparisons can be found in Fig. 1.

**Table 2:** Association between plasma and MRI biomarkers

	MTA		GCA-F		PA		Fazekas		Group differences (Mann-Whitney U)
	Normal	Abnormal	Normal	Abnormal	Normal	Abnormal	Normal	Abnormal	
DLB	*N* = 59	*N* = 33	*N* = 63	*N* = 29	*N* = 47	*N* = 45	*N* = 55	*N* = 34	
GFAP pg/mL, median (IQR)	101.73 (72.14, 161.83)	183.68 (142.69, 261.14)	108.65 (81.25, 174.32)	180.87 (102.54, 256.46)	131.16 (72.14, 189.44)	146.14 (85.19, 195.13)	103.30 (71.66, 167.44)	177.32 (110.64, 264.64)	a, b,c
NfL pg/mL, median (IQR)	19.57 (14.51, 27.94)	29.19 (19.92, 47.70)	19.58 (14.51, 28.07)	29.19 (20.00, 35.76)	19.40 (13.96, 32.34)	23.55 (17.65, 34.06)	18.42 (13.96, 26.87)	27.58 (19.54, 38.55)	a, b,c,
Aβ42/40 , median (IQR)	0.065 (0.058, 0.073)	0.057 (0.051, 0.072)	0.064 (0.056, 0.075)	0.061 (0.055, 0.070)	0.066 (0.057, 0.077)	0.061 (0.052, 0.068)	0.066 (0.058, 0.077)	0.059 (0.050, 0.069)	c, d
pTau231 pg/mL, median (IQR)	9.75 (7.08, 14.00)	12.07 (8.84, 16.48)	9.75 (7.96, 13.77)	11.69 (8.00, 16.51)	10.62 (8.28, 14.83)	9.77 (7.26, 15.87)	10.27 (7.95, 13.23)	9.67 (7.86, 15.88)	
pTau181 pg/mL, median (IQR)	13.23 (9.90, 17.65)	15.01 (12.19, 21.21)	13.47 (10.68, 17.24)	14.40 (11.14, 23.54)	14.24 (11.08, 19.93)	13.67 (10.46, 20.39)	13.47 (10.60, 17.24)	14.66 (11.00, 22.81)	
AD	*N* = 24	*N* = 19	*N* = 31	*N* = 12	*N* = 14	*N* = 29	*N* = 25	*N* = 17	
GFAP pg/mL, median (IQR)	168.59 (107.75, 238.53)	205.66 (143.21, 244.04)	178.39 (121.79, 225.47)	187.50 (135.70, 260.29)	168.59 (118.71, 222.19)	204.76 (132.94, 243.80)	165.07 (97.34, 214.93)	220.08 (173.10, 254.87)	c
NfL pg/mL, median (IQR)	25.48 (17.92, 36.80)	28.00 (21.79, 34.79)	27.93 (18.56, 34.46)	27.65 (18.09, 36.81)	23.40 (16.51, 32.59)	27.93 (22.00, 37.22)	23.03 (17.73, 35.80)	30.09 (24.63, 35.56)	
Aβ42/40, median (IQR)	0.059 (0.052, 0.063)	0.053 (0.048, 0.062)	0.059 (0.052, 0.063)	0.051 (0.047, 0.060)	0.059 (0.047, 0.063)	0.056 (0.050, 0.064)	0.059 (0.050, 0.063)	0.058 (0.049, 0.064)	
pTau231 pg/mL, median (IQR)	14.25 (10.34, 18.37)	13.69 (9.74, 19.99)	14.71 (10.84, 19.43)	12.45 (9.08, 17.44)	14.66 (10.98, 18.22)	13.83 (10.21, 19.80)	14.71 (10.51, 19.80)	12.47 (7.88, 15.36)	
pTau181 pg/mL, median (IQR)	19.62 (15.98, 22.69)	22.23 (17.68, 29.49)	20.38 (17.10, 27.31)	19.49 (12.58, 26.38)	20.53 (15.01, 24.19)	20.18 (16.44, 27.11)	20.68 (16.35, 26.78)	19.62 (14.39, 26.30)	

The table shows medians (interquartile range) for each plasma biomarker separately for patients with normal versus abnormal MRI markers, stratified by DLB and AD groups. a: p-value < 0.05 for MTA; b: p-value < 0.05 for GCA-F; c: p-value < 0.05 for Fazekas; d: p-value < 0.05 for PA. Abbreviations, MTA: medial temporal lobe atrophy; GCA-F: global cortical atrophy scale – frontal subscale; PA: posterior atrophy; DLB: Dementia with Lewy Bodies; AD: Alzheimer Disease; GFAP: glial fibrillary acidic protein; NfL: neurofilament light; Aβ42/40: amyloid β 1–42/ amyloid β 1–40 ratio; pTau231: phosphorylated Tau 231; pTau181: phosphorylated Tau 181

In the DLB group, GFAP and NfL levels were higher in patients with more atrophy in medial temporal lobe (i.e. MTA) and frontal lobe (GCA-F), and a higher cerebrovascular lesion load (Fazekas). Moreover, the Aβ42/40 ratio was lower in DLB patients with more posterior atrophy (PA) and a higher cerebrovascular lesion load (Fazekas). In contrast, pTau231 and pTau181 were not significantly associated with any of the MRI measures.

In the AD group, GFAP levels were higher in patients with a higher cerebrovascular lesion load (Fazekas). Given the smaller size of the AD group compared to the DLB group, we considered effect sizes alongside p-values. Supplementary Table 1 shows a high consistency between p-values and effect sizes. In the AD group, we observed small effect sizes for the following non-statistically significant associations: higher NfL levels with greater cerebrovascular lesion load (Fazekas) (*p* = 0.093, ES = 0.26), lower pTau231 levels with greater cerebrovascular lesion load (Fazekas) (*p* = 0.104, ES=-0.25), and lower Aβ42/40 levels with increased frontal atrophy (GCA-F) (*p* = 0.165, ES=-0.21) (Supplementary Table 1).

### Plasma biomarkers across atrophy subtypes in DLB

**Fig. 2:**
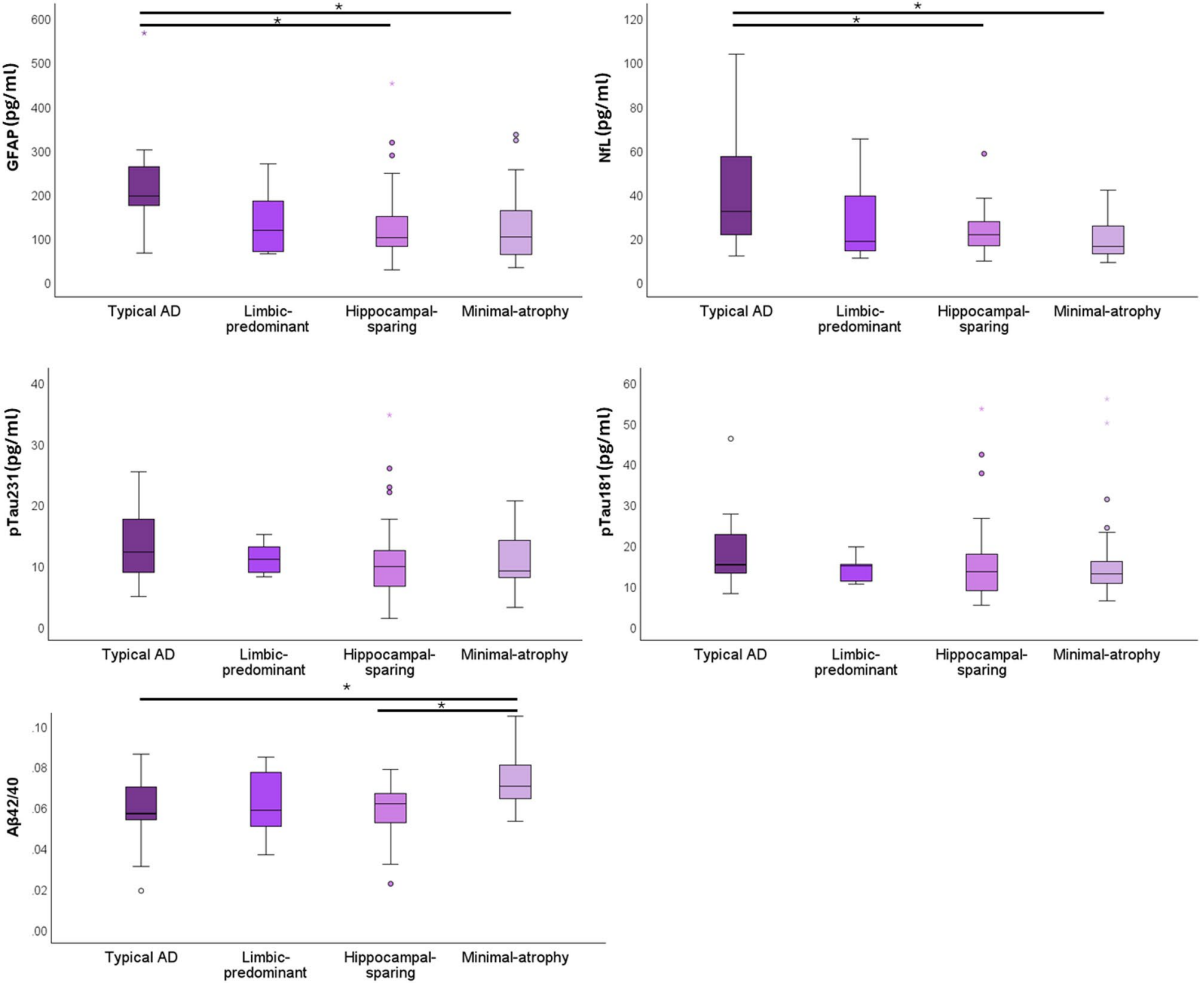
Box-plots for plasma biomarker levels across atrophy subtypes in DLB. *: p-value < 0.005 (Kruskal-Wallis test with the Bonferroni correction). Abbreviations, GFAP: glial fibrillary acidic protein; NfL: neurofilament light; Aβ42/40: amyloid β 1–42/ amyloid β 1–40 ratio; pTau231: phosphorylated Tau 231; pTau181: phosphorylated Tau 181; AD: Alzheimer Disease

Figure 2 and Supplementary Table 2 display the levels of plasma biomarkers across atrophy subtypes (typical AD, limbic predominant, hippocampal-sparing, and minimal-atrophy subtypes) in the DLB group. No differences were found for sex (*p* = 0.738) and MMSE (*p* = 0.154) across atrophy subtypes. However, subtypes differed significantly on age (*p* < 0.001), being minimal atrophy participants younger than typical AD.

We observed statistically significant differences across atrophy subtypes for GFAP, NfL, and Aβ42/40. Specifically, the typical-AD subtype showed higher levels than the hippocampal-sparing and minimal atrophy subtypes for GFAP (*p* < 0.001, *p* < 0.001, respectively) and NfL levels (*p* = 0.035, *p* < 0.001). In addition, the minimal atrophy subtype showed higher Aβ42/40 levels compared with the hippocampal-sparing and the typical AD subtype. When repeating all these analyses correcting for age in ANCOVA, we obtained similar results (Supplementary Table 2).

### The ability of plasma and MRI biomarkers to discriminate DLB from AD

Table 3 and Supplementary Fig. 1 show the ability of plasma and MRI biomarkers to discriminate DLB from AD. Specifically, Table 3 first shows group differences and AUC values, with p-values obtained without any correction. This is followed by group differences and AUC values with p-values obtained with a correction for age and MMSE.

**Table 3:** The ability of plasma and MRI biomarkers to discriminate DLB from AD

	DLB (*n* = 92)	AD (*n* = 43)	Group differences from Mann-Whitney U/Chi square. *p*-value	Discrimination of DLB from AD. AUC-ROC (*p*-value)	Group differences from ANCOVA (Age- and MMSE-corrected results). *p*-value	Discrimination of DLB from AD (Age- and MMSE-corrected results). AUC-ROC (*p*-value)
GFAP pg/mL, median (IQR)	133 (82, 188)	178 (133, 243)	**0.007**	0.66 (**0.001**)	**< 0.001**	0.53 (0.607)
NfL pg/mL, median (IQR)	22 (16, 33)	28 (18, 35)	0.083	0.60 (0.068)	**< 0.001**	0.51 (0.856)
Aβ42/40	0.063 (0.055, 0.073)	0.058 (0.050, 0.063)	**0.002**	0.67 **(< 0.001**)	**0.003**	0.51 (0.894)
pTau231 pg/mL, median (IQR)	10 (8, 15)	14 (10, 19)	**0.004**	0.66 (**0.002**)	**0.002**	0.50 (0.607)
pTau181 pg/mL, median (IQR)	14 (11, 20)	20 (16, 27)	**< 0.001**	0.72 (**< 0.001**)	**0.020**	0.55 (0.401)
MTA (positive, n (%))	33 (35.9%)	19 (44.2%)	0.355	0.55 (0.414)	**0.015**	0.58 (0.183)
GCA-F (positive, n (%))	29 (31.5%)	12 (27.9%)	0.670	0.49 (0.846)	**0.004**	0.51 (0.891)
PA (positive, n (%))	45 (48.9%)	29 (67.4%)	**0.044**	0.59 (0.113)	0.152	0.66 (**0.004**)
Fazekas (positive, n (%))	34 (37%)	17 (39.5%)	0.803	0.52 (0.779)	**< 0.001**	0.50 (0.973)

Medians and interquartile ranges reported for DLB and AD, without any correction. MRI biomarkers were dichotomized as normal and abnormal. Bold denotes statistically significant results (*p* < 0.05). Abbreviations, DLB: Dementia with Lewy Bodies; AD: Alzheimer Disease; GFAP: glial fibrillary acidic protein; NfL: neurofilament light; Aβ42/40: amyloid β 1–42/ amyloid β 1–40 ratio; pTau231: phosphorylated Tau 231; pTau181: phosphorylated Tau 181; MTA: medial temporal lobe atrophy; GCA-F: global cortical atrophy scale – frontal subscale; PA: posterior atrophy

#### Plasma biomarkers alone

All plasma biomarkers showed lower levels in DLB compared to AD patients, except for the Aβ42/40 ratio where AD patients showed lower levels. Group differences were all statistically significant when correcting for age and MMSE.

ROC analyses for each plasma biomarker individually generally showed moderate AUC values, with plasma pTau181 achieving the highest discriminatory performance (AUC 0.72, *p* < 0.001) without any correction. However, the correction for age and MMSE influenced AUC values substantially, with none of the plasma biomarkers discriminating DLB from AD better than chance (*p* > 0.05).

#### Plasma biomarkers combined

We next combined all plasma biomarkers using Random Forest analysis. The model achieved an accuracy of 74.2%. Including age, sex, years of education, and MMSE in the Random Forest model did not improve the discriminatory ability (accuracy 74.6%). The five most relevant variables in the discrimination between DLB and AD were plasma biomarkers pTau181, Aβ42/40 ratio, and pTau231, followed by age and MMSE (supplementary Table 3).

#### MRI biomarkers alone

Regarding group differences for MRI markers, prior correction, there were only statistically significant differences in PA, with a higher proportion of AD patients showing an abnormal score in PA as compared with DLB patients.

None of the MRI measures (MTA, GCA-F, PA, Fazekas) achieved a statistically significant ability to discriminate the two groups prior correction. However, when correcting for age and MMSE, ROC analysis showed that PA could significantly discriminate between DLB and AD (AUC 0.66, *p* = 0.004).

#### MRI biomarkers combined

When combining all MRI biomarkers among them and with demographic variables and MMSE using Random Forest, the model provided an accuracy of 61.0%. MTA and PA were the most contributing variables to the model. However, MRI biomarkers alone without demographic variables and MMSE could not generate a reliable model.

### Combination of plasma and MRI biomarkers with core clinical features to discriminate DLB from AD

Finally, we evaluated the ability of plasma and MRI biomarkers to improve the ability of core clinical features (visual hallucinations, parkinsonism, and cognitive fluctuations) to discriminate DLB from AD. These analyses were based on recurrent Random Forest models combining different type of measures (Table 4). The combination of core clinical features reached an accuracy of 81.8% to discriminate DLB from AD, with a high sensitivity (88.2%) but a low specificity (67.7%). The Random Forest adding MRI biomarkers to the core clinical features provided a similar accuracy (81.6%). PA was the MRI measure achieving the highest importance in the model. On the contrary, the model adding plasma biomarkers to the core clinical features did increase the accuracy (86.6%), with a notable increase of the specificity (78.6%). The Aβ42/40 ratio was the plasma biomarker achieving the highest importance in the model. Adding MRI biomarkers to the model with core clinical features and plasma biomarkers did not change these last results substantially (accuracy = 85.4%, specificity = 79.2%).

**Table 4:** Core clinical features, plasma biomarkers, and MRI visual ratings for the discrimination of DLB from AD

	*N*	Accuracy (95% CI)	Sensitivity (95% CI)	Specificity (95% CI)	Variables contribution (proportion importance)
Clinical features	96	81.8 (73.1, 88.2)	88.2 (78.5, 93.9)	67.7 (50.1, 81.4)	Visual hallucinations: 25.33 Parkinsonism: 18.24 Cognitive fluctuations: 14.74
Clinical features + MRI	98	81.6 (72.8, 88.1)	85.1 (75.3, 91.5)	70.8 (50.8, 85.1)	Parkinsonism: 73.08 Visual hallucinations: 49.10 PA: 38.86 Cognitive fluctuations: 27.05 Fazekas: 16.50 MTA: 14.46
Clinical features + Plasma	97	86.6 (78.4, 92.0)	89.9 (80.5, 95.0)	78.6 (60.5, 89.8)	Parkinsonism: 19.03 Aβ42/40: 18.96 Visual hallucinations: 14.67 Cognitive fluctuations: 9.75 pTau181: 8.47 GFAP: 4.59 NfL: 0.48
Clinical features + Plasma + MRI	96	85.4 (77.0, 91.1)	87.5 (77.9, 93.3)	79.2 (59.5, 90.8)	Parkinsonism: 62.38 Ratio Aβ42/40: 54.42 Visual hallucinations: 44.77 Cognitive fluctuations: 33.17 pTau181: 28.84 GFAP: 14.68 pTau231: 12.76 PA: 8.03

Abbreviations, MRI: magnetic resonance imaging; MTA: medial temporal lobe atrophy; PA: posterior atrophy; GFAP: glial fibrillary acidic protein; NfL: neurofilament light; Aβ42/40: amyloid β 1–42/ amyloid β 1–40 ratio; pTau231: phosphorylated Tau 231; pTau181: phosphorylated Tau 181; CI = confidence interval

## Discussion

This study investigated the association between plasma biomarkers and MRI markers of brain atrophy and CVD in DLB patients compared with AD patients, and tested the ability of plasma and MRI biomarkers to discriminate DLB from AD. The main finding was the significant association of plasma biomarkers of glial activity (i.e., GFAP) and neurodegeneration (i.e. NfL) with atrophy in frontal and medial temporal brain areas as well as CVD in DLB; and the association of amyloid-related pathology biomarkers with atrophy in posterior brain areas and CVD in DLB. These associations were not replicated in the AD group in our study. Plasma biomarkers helped improve the low specificity of core clinical features in discriminating DLB from AD, which may have implications for clinical practice.

Our first aim was to investigate the association of plasma biomarkers with MRI markers of neurodegeneration and CVD co-pathology in DLB, and compare the findings with the associations found in AD. In the DLB group, we found that higher plasma levels of GFAP and NfL were associated with greater brain atrophy in medial temporal and frontal brain areas as well as with a higher cerebrovascular lesion load (Fazekas). GFAP is a biomarker of astrocyte activation while NfL is a biomarker of axonal damage. Astrocyte activation and axonal damage can be caused by diverse pathological processes and so, GFAP and NfL are considered as unspecific biomarkers of neuroinflammation and neurodegeneration, respectively [32, 33]. Previous studies showed the association of brain atrophy with neuroinflammation and unspecific neurodegeneration as assessed with serum and plasma GFAP and NfL in patients with different brain diseases (i.e. traumatic brain injury, multiple sclerosis, etc.) [34–36]. Hence, our current study adds to that literature by demonstrating that the association of brain atrophy with plasma GFAP and NfL also exists in DLB patients. Regarding CVD co-pathology, higher levels of plasma GFAP and NfL were associated with abnormal Fazekas scores in our study. GFAP and NfL have been associated with vascular pathology, suggesting that neuroinflammation may be a key contributor to this association in patients with AD and vascular disease [37, 38]. Further, a previous study reported higher levels of NfL to be associated with brain atrophy and vascular disease in elderly populations with and without cognitive impairment [32]. Our study extends the previous literature by showing similar associations in the DLB population. In our study, most of the associations discussed above were not statistically significant in AD patients, except for the association of plasma GFAP with cerebrovascular lesion load (Fazekas). Our AD group was selected to be comparable in sex to the DLB group, leading to an AD group with more men than usual. That approach makes our AD group a good control for our DLB group, but it may not completely represent AD groups in previous studies, hence the different results [19].

We found that DLB patients with lower plasma levels of Aβ42/40 had greater brain atrophy in posterior brain areas as well as a higher cerebrovascular lesion load. By contrast, we did not find any statistically significant association between pTau plasma biomarkers and MRI markers of atrophy or CVD. A previous study suggested that atrophy in posterior brain areas is the signature atrophy of DLB [6, 39], although atrophy in posterior brain areas may be exacerbated by amyloid and tau co-pathology as assessed with CSF biomarkers [40]. A recent study showed an association of Aβ but not tau with α-synuclein in the CSF [41]. Hence, the association between posterior atrophy and plasma Aβ42/40 in DLB in our current study could reflect the previously shown contribution of amyloid co-pathology towards posterior brain atrophy, perhaps jointly with α-synuclein-related pathology. Previous studies described an association between plasma pTau181 and brain atrophy in AD patients [11, 13]. The absence of significant associations for pTau biomarkers in DLB in our study could be explained by the relatively low proportion of DLB patients with tau pathology, which is around 30% of cases [8]. Another possible explanation is that the contribution of tau co-pathology towards posterior brain atrophy in DLB is less prominent than that of α-syn (and possible amyloid). We found no association between plasma biomarkers of Aβ and MTA, while previous studies showed significant associations between CSF biomarkers of Aβ and MTA both in DLB and AD [40, 42, 43]. A possible explanation is that the contribution of AD co-pathology towards MTA in DLB patients may be captured with CSF biomarkers but not plasma biomarkers [40].

Our second aim was to compare plasma biomarkers across atrophy subtypes in DLB. The motivation for these analyses was to further understand the biological underpinnings of the MRI subtypes previously reported in DLB [6], but also to further assess the potential contributions of AD co-pathology towards the neurodegeneration in DLB. This was particularly relevant in the context of the typical AD atrophy pattern, which is the most common in AD [7]. The only previous study that investigated these subtypes in DLB did not report plasma or CSF biomarkers [6]. The finding of lower Aβ42/40 in typical AD and hippocampal-sparing subtypes compared with the minimal atrophy subtype could reflect the contribution of amyloid co-pathology to these different phenotypes in DLB. In addition, GFAP and NfL showed higher levels in typical AD compared with hippocampal-sparing and minimal atrophy subtypes, which is in line with the widespread and severe neurodegeneration in the typical AD subtype [7]. Previous studies reported the expression of GFAP in pyramidal neurons of the hippocampus in AD [44]. The typical AD subtype has the most severe hippocampal atrophy, but the hippocampus is spared in both minimal atrophy and hippocampal-sparing subtypes [7]. Our finding of higher GFAP levels in typical AD could thus reflect the activity of hippocampal neurons.

Our last aim was to assess the ability of plasma and MRI biomarkers to discriminate between DLB and AD. We started with individual biomarkers. The best individual diagnostic discriminations were observed for plasma pTau181, which achieved an AUC of 0.72 when not corrected for age or MMSE, and the MRI scale for posterior brain atrophy (PA), with an AUC of 0.66. Previous studies evaluated the ability of plasma pTau181, NfL, and GFAP to discriminate among different dementias [45, 46]. These studies found the best accuracies for pTau181 in the discrimination between DLB and AD, while MRI markers were not included. However, a previous study highlighted the importance of correcting these analyses for the effect of variables such as age [47]. Indeed, we found that the ability of plasma biomarkers to discriminate DLB from AD changed considerably when correcting for age (and global cognitive status). In the clinical context, the age of the patient is routinely considered into the diagnostic procedure by clinicians. Therefore, we provided the data prior and after correction for age (and global cognitive status), to inform on the effect of age in our data. For example, for MRI markers, PA showed a substantial increase in its ability to discriminate DLB from AD after the correction for age (and MMSE). This could be related to the finding that PA may be unable to differentiate DLB from AD at older ages [48]. A finding that deserves further investigation is that MTA did not show a significant AUC in our study, despite MTA being traditionally regarded as an important MRI marker to differentiate DLB from AD [4]. Our study lacked neuropathological confirmation and thus, DLB cases with AD co-pathology may have obscured AUC values for MTA.

We next continued with combinations of biomarkers with themselves and with clinical features. A previous study found that the combination of three atrophy measures (i.e. MTA, GCA-F, PA) and Fazekas improved the accuracy obtained for MTA alone in discriminating DLB from AD [49]. In our study, MRI and plasma biomarkers showed a rather limited ability to discriminate DLB from AD individually. However, we found that plasma biomarkers improved baseline diagnostic performance of core clinical features substantially. Specifically, core clinical features of parkinsonism, visual hallucinations, and cognitive fluctuations discriminated DLB from AD with an accuracy of 82% in our study. Sensitivity was 88% but specificity was low (68%). Importantly, plasma biomarkers increased the accuracy up to 87%, mostly positively influencing specificity (increase from 68% up to 79%) without compromising sensitivity (90%). The plasma biomarkers contributing the highest to the discrimination were the Aβ42/40 ratio followed by pTau181, GFAP and NFL. Of note, while parkinsonism was the most discriminating feature, the Aβ42/40 ratio had a higher contribution to the model above and beyond other core clinical features such as visual hallucinations and cognitive fluctuations. The specificity of 79% achieved by this model is superior than the specificity reported in other studies for DLB supportive biomarkers such as FDG-PET (74% specificity) [4]. On the contrary, including MRI in these models did not improve the discrimination of DLB from AD. Altogether, our data suggest that MRI and plasma biomarkers may not aid the discrimination between DLB and AD by themselves, but plasma biomarkers may make a substantial impact to the specificity of core clinical features in the differential diagnosis between these two common neurodegenerative dementias. Since plasma biomarkers are expected to be implemented soon in the clinical routine, this finding may have some clinical implications. A first screening for core clinical features of DLB along with plasma biomarkers may help the subsequent patient flow in specialized clinics, possibly reducing the common delay in DLB diagnosis.

This study has some limitations. Firstly, the sample size was smaller for the AD group than for the DLB group, which mostly involves the associations reported in Table 2. Although this study focused primarily on DLB, the smaller size of the AD group could have led to obtain less significant associations for the AD patients. To handle this issue, we inspected effect sizes in addition to p-values and concluded that the lower number of statistically significant associations in the AD group does not seems to be explained by its smaller group size. Secondly, this study relied on clinical diagnosis, while we did not have access to CSF or PET biomarkers, validated plasma cut-offs for the pTau/Aβ42 ratio, or neuropathological diagnostic confirmation. Therefore, we could not stratify the DLB group based on AD biomarkers. This limits the possibility to distinguish between “pure” DLB and mixed DLB-AD profiles, which could potentially influence the reported associations between plasma biomarkers and MRI measures. The focus of this study on plasma biomarkers however provides some insight into the underlying pathophysiology. Thirdly, RBD is an important core clinical feature in the 2017 update of the diagnostic criteria for DLB [4]. However, RBD data was not available in our study and was not included in the Random Forest models. Previous publications showed that the addition of RBD primarily increased the lower sensitivity of the 2005 diagnostic criteria [50, 51]. Hence, the lack of RBD data in our study may have had a minor impact since the sensitivity of the remaining core clinical features was rather high (i.e. 88.2%). Finally, the age differences between the DLB and AD groups may make results interpretation difficult. However, we leveraged this situation as it may reflect actual differences found in clinical practice, which often contribute to diagnostic decisions. In clinical practice, DLB patients are often younger than AD patients, like in our study. Hence, this finding may increase the representativity and clinical generalization of our results. By reporting uncorrected and age-corrected results we elaborated on the impact of age on the discrimination between DLB and AD groups, which is a topic that has not been documented enough in previous studies. The age also reflects time and disease progression and, therefore, the age is biologically involved in the disease process we are investigating instead of just being a mere confounder.

## Conclusions

This study demonstrates that plasma biomarkers of AD co-pathology, glial processes and unspecific neurodegeneration partly explain the brain atrophy and cerebrovascular disease-related neurodegeneration observed in DLB patients. While the investigated plasma and MRI biomarkers may not resolve the differential diagnosis between DLB and AD by themselves, the combination of plasma biomarkers with core clinical features helped increase the diagnostic specificity from 68% up to 79%, with a sensitivity of 90%. Including plasma biomarkers in the clinical work-up may help expediting the referral of patients for specialized assessment and, thus, reduce delays in DLB diagnosis.

## Supplementary Information

Below is the link to the electronic supplementary material.

Supplementary Material 1

## Data Availability

The datasets used and/or analysed during the current study are available from the corresponding author on reasonable request.

## Abbreviations

Aβ: Amyloid β
AD: Alzheimer’s Disease
CT: Computed Tomography
CVD: Cerebrovascular disease
CSF: Cerebrospinal Fluid
DaTSCAN: Dopamine transporter Scan
DLB: Dementia with Lewy Bodies
GCA-F: Global Cortical Atrophy scale – Frontal subscale
GFAP: Glial Fibrillary Acidic Protein
MIBG: 123Iodine-metaiodobenzylguanidine
MMSE: Mini-Mental State Examination
MRI: Magnetic Resonance Imaging
MTA: Medial Temporal lobe Atrophy
NfL: Neurofilament Light
PA: Posterior Atrophy
PET: Positron Emission Tomography
p-Tau: Phosphorylated Tau
RBD: Rapid eye movement sleep Behaviour Disorder
