# Supplementary material for: The association between plasma and MRI biomarkers in dementia with lewy bodies
Source: Alzheimers Res Ther. 2025 Aug 22;17:197. doi: 10.1186/s13195-025-01848-x (PMC12374387; doi:10.1186/s13195-025-01848-x)
Supplement: Supplementary file 1 — Supplementary Material 1 [file 13195_2025_1848_MOESM1_ESM.docx]

**Supplementary Table 1. Effect sizes for plasma and MRI biomarkers in AD and DLB.**

|  | | MTA  p-value  (Effect size) | GCA-F  p-value  (Effect size) | PA  p-value  (Effect size) | Fazekas  p-value  (Effect size) |
| --- | --- | --- | --- | --- | --- |
| DLB | GFAP | **<0.001**  **(0.40)** | **0.010**  **(0.25)** | 0.740  (0.05) | **0.001**  **(0.34)** |
|  | NfL | **0.002**  **(0.32)** | **0.020**  **(0.24)** | 0.161  (0.15) | **0.001**  **(0.35)** |
|  | Aβ42/40 | 0.099  (-0.17) | 0.261  (-0.12) | **0.049**  **(-0.21)** | **0.009**  **-0.28** |
|  | pTau231 | 0.060  (0.19) | 0.214  (0.13) | 0.507  (-0.07) | 0.735  (0.04) |
|  | pTau181 | 0.084  (0.18) | 0.161  (0.15) | 0.836  (-0.02) | 0.194  (0.14) |
| AD | GFAP | 0.304  (0.16) | 0.512  (0.10) | 0.641  (0.07) | **0.025**  **(0.35)** |
|  | NfL | 0.625  (0.07) | 0.947  (0.01) | 0.204  (0.19) | 0.093  **(0.26)** |
|  | Aβ42/40 | 0.212  (-0.19) | 0.165  **(-0.21)** | 0.917  (0.02) | 0.929  (0.01) |
|  | pTau231 | 0.950  (-0.01) | 0.328  (-0.15) | 0.927  (0.02) | 0.104  **(-0.25)** |
|  | pTau181 | 0.240  (0.18) | 0.500  (-0.11) | 0.885  (0.02) | 0.597  (-0.08) |

Interpretation of effect sizes as 0.2 small effect, 0.5 medium, and 0.8 large. Bold denotes statistically significant results (p<0.05). Abbreviations, MTA: medial temporal lobe atrophy; GCA-F: global cortical atrophy scale – frontal subscale; PA: posterior atrophy; DLB: Dementia with Lewy Bodies; AD: Alzheimer Disease; GFAP: glial fibrillary acidic protein; NfL: neurofilament light; Aβ42/40: amyloid β 1-42/ amyloid β 1-40 ratio; pTau231: phosphorylated Tau 231; pTau181: phosphorylated Tau 181.

**Supplemantary Table 2**. **Plasma biomarker levels across atrophy subtypes in DLB.**

|  | **Typical AD**  **(n=23)** | **Limbic predominant (n=10)** | **Hippocampal-sparing (n=35)** | **Minimal-atrophy (n=24)** | **Uncorrected**  **p-value** | **Posthoc** | **Age-corrected (ANCOVA) p-value** |
| --- | --- | --- | --- | --- | --- | --- | --- |
| GFAP,  pg/mL  Median (IQR) | 196  (172, 267) | 118  (69, 197) | 102  (81, 151) | 104  (62, 163) | **<0.001** | a,b | **<0.001** |
| NfL,  pg/mL  Median  (IQR) | 32  (22, 58) | 19  (14, 39) | 22  (16, 28) | 16  (13, 27) | **<0.001** | a,b | **<0.001** |
| Aβ42/40  Median  (IQR) | 0.057  (0.054, 0.070) | 0.058  (0.049, 0.077) | 0.062  (0.052, 0.067) | 0.070  (0.063, 0.082) | **0.005** | a,c | **<0.001** |
| pTau231,  pg/mL  Median  (IQR) | 12  (9, 18) | 11  (9, 13) | 10  (6, 12) | 9  (8, 14) | 0.288 |  | 0.102 |
| pTau181,  pg/mL  Median  (IQR) | 15  (13, 23) | 15  (11, 16) | 14  (8, 18) | 13  (11, 16) | 0.264 |  | 0.388 |

a: posthoc differences between Typical AD and minimal atrophy; b: posthoc differences between Typical AD and hippocampal-sparing; c: posthoc differences between hipocampal-sparing and minimal atrophy. Bold denotes statistically significant results (p<0.05). Abbreviations, AD: Alzheimer Disease; IQR: interquartile range; GFAP: glial fibrillary acidic protein; NfL: neurofilament light; Aβ42/40: amyloid β 1-42/ amyloid β 1-40 ratio; pTau231: phosphorylated Tau 231; pTau181: phosphorylated Tau 181. Bold denotes statistically significant results (p<0.05).

**Supplementary Table 3.** Random Forest models for plasma biomarkers, MRI visual ratings and demographic variables.

|  | N | Accuracy  (95% CI) | Sensitivity (95% CI) | Specificity  (95% CI) | Variables contribution  (prop imp) |
| --- | --- | --- | --- | --- | --- |
| MRI + Demographics | 118 | 61.0  (52.0, 69.3) | 68.4  (58.5, 76.9) | 30.4  (15.6, 50.9) | MTA: 29.01  PA: 17.14  MMSE: 16.90  Age: 8.45  Fazekas: 4.25 |
| Plasma | 132 | 74.2  (66.2, 80.9) | 78.6  (69.5, 85.5) | 61.8  (45.0, 76.1) | pT7au181: 18.41  Ratio Aβ42/Aβ40: 15.15  pTau231: 5.23  NfL: 2.74  GFAP: 0.74 |
| Plasma + Demographics | 118 | 74.6  (66.0, 81.6) | 78.0  (68.5, 85.3) | 63.0  (44.2, 78.5) | pTau181: 18.17  Ratio Aβ42/Aβ40: 13.24  pTau231: 6.04  Age: 5.56  MMSE: 4.40  Education (years): 3.81  NfL: 3.35  GFAP: 2.60  Sex: 1.56 |
| Plasma + MRI | 128 | 78.1  (70.2, 84.4) | 81.1  (72.0, 87.7) | 69.7  (52.7, 82.6) | pTau181: 61.29  Ratio Aβ42/Aβ40: 46.54  pTau231: 15.19  GFAP: 6.06  MTA: 4.32 |
| Plasma + MRI + demographics | 115 | 73.0  (64.3, 80.3) | 77.3  (67.5, 84.8) | 59.3  (40.7, 75.5) | pTau181: 41.23  Ratio Aβ42/Aβ40: 34.49  pTau231: 23.83  Age: 9.92  GFAP: 7.93  MMSE: 5.51  Education (years): 5.13  PA: 3.53  MTA: 3.40  GCA: -0.30 |

Abbreviations, MTA: medial temporal lobe atrophy; GCA-F: global cortical atrophy scale – frontal subscale; PA: posterior atrophy; DLB: Dementia with Lewy Bodies; AD: Alzheimer Disease; GFAP: glial fibrillary acidic protein; NfL: neurofilament light; Aβ42/40: amyloid β 1-42/ amyloid β 1-40 ratio; pTau231: phosphorylated Tau 231; pTau181: phosphorylated Tau 181; Prop imp: proportion of importance, which measures the contribution of each variable to the model’s prediction. Higher prop imp values mean a greater contribution of the variable to the model.

**Suplementary Figure 1.** ROC curves for the ability of plasma and MRI biomarkers to discriminate DLB from AD. The figure shows the AUC-ROC without correcting for age and MMSE. Abbreviations, MTA: medial temporal lobe atrophy; PA: posterior atrophy; GCA-F: global cortical atrophy scale – frontal subscale; GFAP: glial fibrillary acidic protein; NfL: neurofilament light; Aβ42/40: amyloid β 1-42/ amyloid β 1-40 ratio; pTau231: phosphorylated Tau 231; pTau181: phosphorylated Tau 181; ROC: Receiver operating characteristic.
